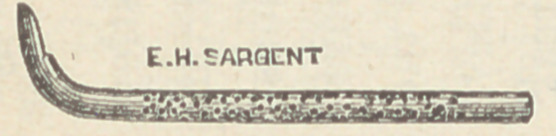# A Modification of the Style

**Published:** 1878-10

**Authors:** S. O. Richey

**Affiliations:** Chicago


### A MODIFICATION OF THE STYLE.

S. O. Richey, M. D., Chicago.

It is unnecessary to reiterate recognized facts with regard to the persistence of inflammation of the lachrymal sac and the nasal duct, and the obstinate resistance of strictures of the duct, to treatment. Forcing water through the passage is a method which, in some cases, has given good results, but chiefly in those in which the obstruction is due to inflammation of short duration. When structural changes have taken place in the duct with the formation of strictures, the treatment just mentioned is of small value, and more radical measures are called for.

The frequent passing of probes to dilate the strictures generally produces so much irritation as to keep up the trouble. The hollow style has enabled us to obtain better results by keeping open the passage, while it adds to the patient's comfort by allowing the tears to pass in the direction of their natural channel. When the style occupies the duct there is no chance of forcing any fluid through the duct itself by the side of the tube, and therefore, to treat the lining membrane of the sac and duct, the instrument must be partially or wholly removed, that the canal may be washed out, and the applications made.

The withdrawal and replacement of the style has much the same effect as passing the probe, and the object of the present modification is to obviate the necessity for removing it frequently, while by means of a small syringe it may be cleansed, and the requisite application made to the affected surface.

The change which I have made consists merely of numerous perforations, allowing free communication between the inside and the outside of the tube, thereby exposing as much as possible of the lining membrane of the canal while the style is in position.

Sufficient only of the metal forming the tube is left to maintain the required shape of the instrument, and thus to preserve the dilation of the strictures.

The wood cut represents a style the size of No. 8, Bowman's probe; its usual length is about 3.33 centimetres. The perforations answer a double purpose, viz: they permit any secretion from the membrane to pass into the tube, whence it may be washed into the nostril, thus preventing its accumulation in the sac and duct, and the irritation which its presence there would naturally cause; they permit the medicated fluid to reach the membrane, which has already been freed from the secretion; both of which objects may be accomplished without withdrawing the style. After a little practice the patient may be able to perform the service for himself, which is a special advantage, when he can not remain constantly under the supervision of the surgeon.

The style may be made to correspond in size with any of Bowman's probes, from No. 5 to No. 8, inclusive, and may be procured of Sargent & Co.

## Figures and Tables

**Figure f1:**